# Editorial

**Published:** 1904-01-15

**Authors:** 


### EDITORIAL.

One of our esteemed contemporaries reprints an article by a teacher in a dental college on Dental Education, and commends it as worthy of thoughtful consideration by those who are interested in the changes of educational standards now under discussion. Some things in the article quoted are well worth pondering, but there is so much that is exaggerated or carelessly stated that the article looses much of the force it otherwise would have. For instance, the author says, "there is no question in the mind of the writer that we have ten times too many colleges, and not one tenths enough teachers, and indeed not enough departments in the present curriculum." As we now have fifty Dental Colleges, we should reduce the number to five to suit this writer, and as we now have in round numbers five hundred teachers, we should increase the number to five thousand. The writer fails to specify the muliplication he would make in subjects for the curriculum, but if in proportion we should increase at least to double the present number, or say about forty. If this were done we should have to extend the teaching time to at least five or six years and teach eight or nine months each year. How absurd! Think of trying to teach seven thousand students with five thousand teachers in five school houses. The same article says: "Of ideals, ambitions or aspirations the present teachers of technical subjects have none." There is a lot more of such unfair criticism that space or inclination give us no opportunity to mention. Most of our teachers are making commendable efforts and at great pecuniary sacrifice to help on educational affairs.

